# Reliability‑based probabilistic numerical plastically limited analysis of reinforced concrete haunched beams

**DOI:** 10.1038/s41598-023-29930-0

**Published:** 2023-02-15

**Authors:** Sarah Khaleel Ibrahim, Majid Movahedi Rad

**Affiliations:** Department of Structural and Geotechnical Engineering, Széchenyi István University, Gyor, 9026 Hungary

**Keywords:** Engineering, Civil engineering

## Abstract

This research proposes a novel method that considers the optimal reliability-based design of reinforced concrete haunched beams subjected to probabilistic concrete properties and complementary strain energy values. The theory is applied twice, once to evaluate a deterministic solution, where the plastic behaviour is constrained by the complementary strain energy of residual stresses induced within steel bars. Secondly, the same method is considered for a probabilistic solution where the concrete characteristics—compressive strength and modulus of elasticity—and complementary strain energy value vary randomly. The reliability index acts as a bound for the solution. It is important to mention that the model utilised in this publication is derived from recent research after being calibrated using Abaqus. This work represents an extension of that recent research that exclusively considered deterministic work. This research led deterministically to new estimates for the complementary strain energy, which will be interpreted as reflecting the behaviour of plastic thresholds and quantifying the associated loads. Afterwards, uncertainty is studied when probability interferes, showing various load values and damage in concrete and steel when the complementary strain energy and concrete properties are probabilistically evaluated, giving a less reliable solution as the load reduces. These effects are reflected in the varying plastic behaviour of the investigated haunched beams.

## Introduction

Haunched beams or non-prismatic are elements with different cross-section features from one part to the other, with no straight axis. Several researchers were interested in researching non-prismatic concrete beams because they could save concrete and steel if used to substitute similar strength prismatic sections.

According to Jolly and Vijayan^1^, in 2015, when the span increases, beams become uneconomical because of an increase in depth. Non-prismatic beams or haunched beams are a worthy option in this circumstance. Their study involved seismic analysis of RC frames taking into account both linear and haunch beams and the structural behaviour of reinforced concrete haunched beams using ANSYS and ETABS. Orr et al.^2^ presented research on non-prismatic RC beams designed with three distinct methods indicating that the principles of certain design codes may cause an unconservative shear design for non-prismatic segments. Hou et al.^3^ investigated the mechanism of shear resistance of reinforced concrete haunched beams (RCHBS) with different indicators (location of haunched parts, concrete cover thickness, existence of stirrups, and tensile reinforcement layout). The outcomes showed that the bending location of the tensile reinforcement bars and the various preparation of the tensile reinforcement bars hugely affected the crack propagations that induced the variation. Godnez-Domnguez et al.^4^ showed the outcomes of numerous nonlinear finite element modelling for many simply supported reinforced concrete haunched beams, wherein simpler nonlinear models with the involvement of longitudinal steel reinforcement and stirrups were studied using SAP2000. Tena-Colunga et al.^5^ also investigated reinforced concrete beams in the case of prismatic and haunched, built and evaluated to collapse in shear with significant damage.

Optimisation, on the other hand, is provided to identify the best-suited candidate from a collection of accessible possibilities without explicitly enumerating and analysing all feasible alternatives. Consequently, various studies on the optimum solution for reinforced concrete (RC) structures have been conducted. Azam et al.^6^ highlighted the use of numerous optimisation methodologies in attaining a cost-effective design, underlining the usefulness of the Solver tool in MS Excel as an easy-to-use choice for the design and optimisation of simply supported RC. Whereas Shariat et al.^7^ performed an optimisation and sensitivity study on rectangle reinforced concrete (RC) beams utilising the computationally Lagrangian Multiplier Method (LMM) as programming optimisation computer software. By using the particle swarm optimisation (PSO) approach, Hanoon et al.^8^ suggested an energy-absorbing model for forecasting the influence of loading rates, concrete strength properties, shear span-to-depth ratio, and transverse and longitudinal reinforcement ratio on (RC) beams. Perera and Vique^9^ also described a technique for automatically constructing optimum strut-and-tie models to design reinforced concrete beams. The optimum model is created by solving an optimal solution with evolutionary algorithms. Sharafi et al.^10^ developed a new cost-optimisation approach for geometric layout issues that takes both cost factors and dynamic responses to create an optimised design while maintaining the appropriate dynamic response of RC beams.

Because the parameters used in structural verifications are sensitive to variability and thus random, structural codes often standardise safety by offering sets of deterministic verifications calibrated by code authors using probabilistic ideas. To achieve this calibration, partial safety factors are defined. These factors ensure that the probabilities of noncompliance with deterministic safety verifications closely resemble the target probability of failure (*Pf*) based on failure consequences. Kappos et al. explored the ductility and strength of reinforced concrete beams and column cross-sectional areas with various geometries and confinement layers. Simulating materials' properties as random variables and evaluating their effect on section behaviour and attitude using fiber modelling and the Response Surface Method^11^. Additionally, Sykora et al. employed proper modelling of material characteristics, geometrical parameters, and uncertainties connected to an adapted model for the failure process under consideration to anticipate load-bearing capacity^12^. Besides that, Yu et al. explored the variance implicit in the behaviour of RC frame structures to bridge over a column loss, carrying uncertainty in gravity loads, properties of the material, and construction geometries into consideration. Sensitivity and uncertainty analyses have been conducted to provide the impact and relevance of the uncertain parameters on RC frames to hold the total collapse^13^.

Thus, design approaches are recommended to account for the randomness of material properties and load situations where the mechanical properties of materials cannot be assessed deterministically, which is especially relevant in the case of RC structures. When these characteristics' unpredictability is considered, the structural reaction takes on a probabilistic form, necessitating a reliability study. Marano^14^ developed an efficient alternative method for time-dependent reliability evaluation of reinforced concrete beams subjected to pitting corrosion in which probabilistic and non-probabilistic properties exist. Using this unique method, it was possible to get a versatile tool to aid decision-makers in planning maintenance interventions. Seghier et al.^15^ studied the use of a structural reliability methodology, especially the three-term conjugate map (TCM) relying on the first-order reliability method (FORM), for the multi-state assessment of corroded RC beams. Stewart^16^ also developed a probabilistic model to evaluate immediate shrinkage deflections and creep. Monte Carlo simulation is used to evaluate deflections and probabilities of serviceability failure for reinforced concrete beams sized to meet serviceability specifications on span-to-depth ratio.

Several structures and elements have residual stresses, and residual stress in various components can be accurately assessed using many methods. Thus, designing modules and structural elements and testing their reliability in service requires residual stress analysis^17^. Complementary strain energy within elements has been shown to control residual stresses and plastic behaviour. Calculating residual force complementary strain energy can quantify design uncertainties in manufacturing, strength, and geometry and provide a reliability-based extended-limit design problem^18^. Steel frames, piles, and trusses were introduced in this theory^19–21^. All recent studies have focused on steel structures; however, Rad et al.^22^ adapted this theory deterministically to reinforced concrete structures. Then, a probabilistic study considering reliability design was presented in this research.

This study relies on prior research^22^, which developed a novel numerical (optimization) model for managing the plastic behaviour of reinforced concrete haunched beams by leveraging the complementary strain energy of residual forces inside steel reinforcing bars. The optimal elasto-plastic analysis and design of haunched reinforced concrete beams were applied to optimization problems with varying objective functions, such as finding the maximum loading or the minimum volume of the steel used to reinforce the beams. The constraints on the complementary strain energy of the residual internal forces of the steel bars limit plastic deformations. The optimization problems showed beams migrating from elastic to elasto-plastic to fully plastic under varied complementary strain energy limit values.

This investigation conducts a numerical analysis of haunched beams made of reinforced concrete by applying a reliability design. Adding to the previous study, the properties of the used concrete material (compressive strength and modulus of elasticity) and the allowable complementary strain energy values are probabilistic values with mean and standard deviation. At the same time, the reliability index is considered a limitation index and the complementary strain energy inside steel bars is acquired to indicate plastic behaviour. Giving a probabilistic analysis would impact the initiated internal steel stresses, which then added to the prior study's findings. Applying a probabilistic analysis to concrete characteristics will convert this impact into complementary strain energy. Manifesting varied plastic behaviour in the examined haunched beams with the cooperation of the reliability index value specifies the reliability level and helps to understand the uncertainty effect on the constructed elements and damage percentages inside the concrete and steel bars. Notably, the numerical calibration and probabilistic analysis were performed by integrating Abaqus with the authors' own code.

In the deterministic solution, all characteristics are constant. However, different complementary strain energy values were picked, revealing that it affects the affiliated load values, resulting in changed extensive concrete and steel damage. However, probabilistic cases show the effect of reliability index on the strength and damage of the models proving that the reliability index is valuable as a limitation value indicating failure probability. Also, the randomness of probabilistic values reflects material deviation and its effect on structural behaviour. It is essential to understand how uncertainties affect structure strength and damage, as actual structures are uncertain.

A detailed description of the theory of restricted residual plastic deformation in Section "Theory of limiting residual plastic deformation in rebar" follows this introduction. In Section "The problem of reliability-based design", the general concepts of reliability-based design are clarified. Section "Optimization solutions" also details the optimization process, while details of model validation are presented in section "Model validation details". In addition, results and discussions are illustrated in section "Results and discussions". Lastly, the conclusions are presented in Section "Conclusions".

## Theory of limiting residual plastic deformation in rebar

The theory presented in this section was applied and developed by different previous studies^18–22^, where an appropriate computational technique was established so that the complementary strain energy of residual forces (*W*_*p*_) can be recognised as a comprehensive assessment of the plastic behaviour of materials^23–25^ when such energy amount limits are required to regulate residual deformations. This complementary strain energy is generated by residual forces, which are described below:

1 $$W_{p} = \frac{1}{{2{\text{E}}}}{ }\mathop \sum \limits_{{{\text{i}} = 1}}^{{\text{n}}} \frac{{l_{i} }}{{A_{i} }} N_{i}^{{R^{2} }} \le W_{p0}$$

for which *W*_*p*0_ is the maximum allowable energy that can be used to calculate *W*_*p*_ from the structure's elastic strain energy^23^ of the structure. Additionally, $N_{i}^{R}$ is the residual force of the bar members, E is Young's modulus of the bar material, and $l_{i} ,{ }\left( {i = 1,{ }2, \ldots ,n} \right)$ is the member length of the rebar elements, $A_{i} , \left( {i = 1,{ }2, \ldots ,n} \right){ }$ is the rebar elements' cross-section area. A limit value *W*_*p*0_ is introduced for plastic deformations of rebar in Eq. (1). Further, the inner plastic force *N*^*pl*^—which will arise when the load *P*_0_ is applied- and the elastic internal force − *N*^*el*^ characterise the residual forces *N*^*R*^ which remain in the structure after unloading.

2 $$N^{R} = N^{pl} - N^{el}$$

where

3 $$N^{el} = F^{ - 1} G^{T} K^{ - 1} P_{0} .$$

Matrix *F* stands for flexibility, Matrix *G* for geometry, and Matrix *K* for rigidity in this context.

## The problem of reliability-based design

The failure is found to be $X_{R} \le X_{S}$, which is the fundamental concept of reliability being applied. Let's pretend there are two uncorrelated random variables, *X*_*R*_ and *X*_*S*_, and that their respective probability density functions, *f*_*R*_(*X*_*R*_) and *f*_*S*_(*X*_*S*_), are given below. The likelihood of failure is determined by the formulae below^26–28^:

4 $$P_{f} = { }P\left[ {X_{R} \le X_{S} } \right] = { }\mathop {\iint }\limits_{{X_{R} \le X_{S} }}^{{}} f_{R} { }\left( {X_{R} } \right)f_{S} { }\left( {X_{S} } \right)dX_{R} dX_{S} .$$

Hence *P*_*f*_ represents the failure probability. This issue may be summarized in terms of the so-known bound state function, which is defined as:

5 $$g\left( {X_{R} ,X_{S} } \right) = X_{R} - X_{S} .$$

Taking into account that $g{ } \le { }0$ represents the failure domain *D*_*f*_. Accordingly, the failure probability *P*_*f*_ is expressed by:

6 $$P_{f} = F_{g} \left( 0 \right).$$

where *F*_*g*_(0) is the cumulative distribution function of the limit state surface. Additionally, *P*_*f*_ can be calculated by:

7 $$P_{f} = \mathop \smallint \limits_{{g\left( {X_{R} ,X_{S} } \right) \le 0}}^{{}} f\left( X \right)dX = \mathop \smallint \limits_{{D_{f} }}^{{}} f\left( X \right)dX.$$

For the sake of simplicity, this study assumes a Gaussian distribution for the constraint for the value of the complementary strain energy of the residual forces, with a specified mean value of $\overline{W}_{po }$ and standard deviation of *σ*_*w*_, to account for the inherent uncertainties in the data.

In addition, the Monte Carlo sampling method is taken into account for calculating the reliability index (*β*), which is based on the probability of failure (*P*_*f*_). Producing realizations *x* according to a random vector *X* of the probability density function *f*_*X*_(*x*) is central to the Monte Carlo method (x). Therefore, *P*_*f*_ can be obtained by determining the number of total points within the failure domain regarding the number of completely generated points. By introducing an indicator function of *D*_*f*_, this concept can be stated as:

8 $$\chi_{{D_{f} }} \left( x \right) = \left\{ {{ }\begin{array}{*{20}c} {1{ }\,if\,\,{ }x \in { }D_{f} } \\ {0{ }\,if\,{ }x \notin { }D_{f} } \\ \end{array} } \right\}.$$

By rewriting Eq. (7):

9 $$P_{f} = \mathop \int \limits_{ - \infty }^{ + \infty } { }.{ }.{ }.{ }\mathop \int \limits_{ - \infty }^{ + \infty } \chi_{{D_{f} }} \left( x \right)f_{X} \left( x \right)dx$$

where $\chi_{{D_{f} }} \left( X \right)$ is two-points distribution random variable.

10 $${\mathbb{P}}\left[ {{ }\chi_{{D_{f} }} \left( X \right) = 1} \right] = { }P_{f}$$

11 $${\mathbb{P}}\left[ {{ }\chi_{{D_{f} }} \left( X \right) = 0} \right] = { }1 - P_{f}$$

where $$P_{f} = {\mathbb{P}}\left[ {{ }X \in { }D_{{f{ }}} } \right]$$.

To compute the mean ${\mathbb{E}}$ and variance ${\mathbb{V}}ar$ of the random variable $\chi_{{D_{f} }} \left( X \right)$:

12 $${\mathbb{E}}\left[ {\chi_{{D_{f} }} \left( X \right)} \right] = 1 \cdot P_{f} + 0 \cdot \left( {1 - P_{f} } \right) = P_{f}$$

13 $${\mathbb{V}}ar\left[ {\chi_{{D_{f} }} \left( X \right)} \right] = {\mathbb{E}}\left[ {\chi_{{D_{f} }}^{2} \left( X \right)} \right] - ({\mathbb{E}}\left[ {\chi_{{D_{f} }} \left( X \right)} \right])^{2} = P_{f} - P_{f}^{2} = P_{f} \left( {1 - P_{f} } \right).$$

In order to estimate *P*_*f*_ using the Monte Carlo method, we use the following formula:

14 $${\hat{\mathbb{E}}}\left[ {\chi_{{D_{f} }} \left( X \right)} \right] = \frac{1}{Z}\mathop \sum \limits_{z = 1}^{Z} \chi_{{D_{f} }} (X^{\left( z \right)} ) = \hat{P}_{f}$$

where $X^{\left( z \right)}$ is a representation for a set of independent random vectors with random sample number *Z* ($z = 1, \ldots ,Z$), and *f*_*X*_(*x*).

Interestingly, in probabilistic models, bound on the complementary strain energy is treated as a random variable. As a result, it has a mean value and a standard deviation. Furthermore, it follows the Gaussian distribution with a mean ${\mathbb{E}}$ and a variance ${\mathbb{V}}ar$. The estimator's mean and variance values are then easily computed as follows:

15 $${\mathbb{E}}\left[ {\hat{P}_{f} } \right] = \frac{1}{Z}\mathop \sum \limits_{z = 1}^{Z} {\mathbb{E}}\left[ {\chi_{{D_{f} }} \left( {X^{\left( z \right)} } \right)} \right] = \frac{1}{Z}ZP_{f} = P_{f}$$

16 $${\mathbb{V}}ar\left[ {\hat{P}_{f} } \right] = \frac{1}{{Z^{2} }}\mathop \sum \limits_{z = 1}^{Z} {\mathbb{V}}ar\left[ {\chi_{{D_{f} }} \left( {X^{\left( z \right)} } \right)} \right] = \frac{1}{{Z^{2} }}ZP_{f} \left( {1 - P_{f} } \right) = \frac{1}{Z}P_{f} \left( {1 - P_{f} } \right).$$

The reliability constraint then can be presented by expressing (*β*)^29,30^ as:

17 $$\beta_{target} - \beta_{calc} \le 0$$

where *β*_*calc*_ is the reliability index generated for each iteration, and when it exceeds a specific value which is the target value *β*_*target*_ the process will be ended as this constraint is fulfilled.

For determining *β*_*target*_ and *β*_*calc*_, the following terms are used:

18 $$\beta_{target} = - \Phi^{ - 1} \left( {P_{f,target} } \right)$$

19 $$\beta_{calc} = - \Phi^{ - 1} \left( {P_{f,calc} } \right)$$

therefore, the inverse of the normal distribution function, herby Φ^−1^, is the truncated normal distribution. Concrete parameters such as compressive strength ${\mathop{f}\limits^{\prime}}_{c }$ and modulus of elasticity *E*_*c*_ are considered variables in this study.

The reliability index is considered a limitation index inside the programming code that controls the termination of the problem by limiting the complementary strain energy value *W*_*p*_; after that, the corresponding load, deflection and complementary strain energy were obtained.

## Optimization solutions

Nonlinear optimization is used to find the maximum plastic loading *F*^*pl*^ that can be applied to the haunched beams without resorting to iterative updates of the constitutive components. Here are the equations considered by the deterministic solution, where *A*_*i*_ and *l*_*i*_ represent the cross-section and length of each element^23,31^.

20a $$Max.{ } \to { }F^{pl}$$

20b $$Subjected to: N^{el} = { }F^{ - 1} GK^{ - 1} P_{0}$$

20c $$- \overline{{N^{pl} }} \le N^{Pl} \le \overline{{N^{pl} }}$$

20d $$\frac{1}{{2{\text{E}}}}{ }\mathop \sum \limits_{{{\text{i}} = 1}}^{{\text{n}}} \frac{{l_{i} }}{{A_{i} }} N_{i}^{{R^{2} }} \le W_{p0} .$$

21a $$u - u_{o } < 0$$

The elastic fictitious internal normal forces are calculated in Eq. (20b); however, Eq. (20c), where *N*^*pl*^ the ultimate plastic limit load, displays is the lower and upper-plastic limit conditions. Additionally, as a global measure of the structure's plastic behaviour, the complementary strain energy of residual forces used to govern plastic deformations of steel bars is depicted by boundary Eq. (20d).

While Eq. (21a) shows the deflection condition, where *u* represents the determined deflection value through the optimization process while *u*_0_ is the allowable deflection value. For a deterministic solution, it is adequate to assume that the plastic deformations are under control as long as the calculated value of the complementary strain energy of the residual forces is less than or equal to the bound for the magnitude of the allowable complementary strain energy of the residual forces. The solution is terminated once the allowable complementary strain energy *W*_*p*0_ is exceeded.

On the other hand, to study the probabilistic solution, Eq. (20d) is replaced by Eq. (17) $\beta_{target} - \beta_{calc} \le 0$, announcing that the termination condition is then controlled by the probabilistic complementary strain energy randomly chosen and allowed for each iteration while considering the suitable mean value $\overline{W}_{po }$ and standard deviation *σ*_*w*_.

**Figure 1:**
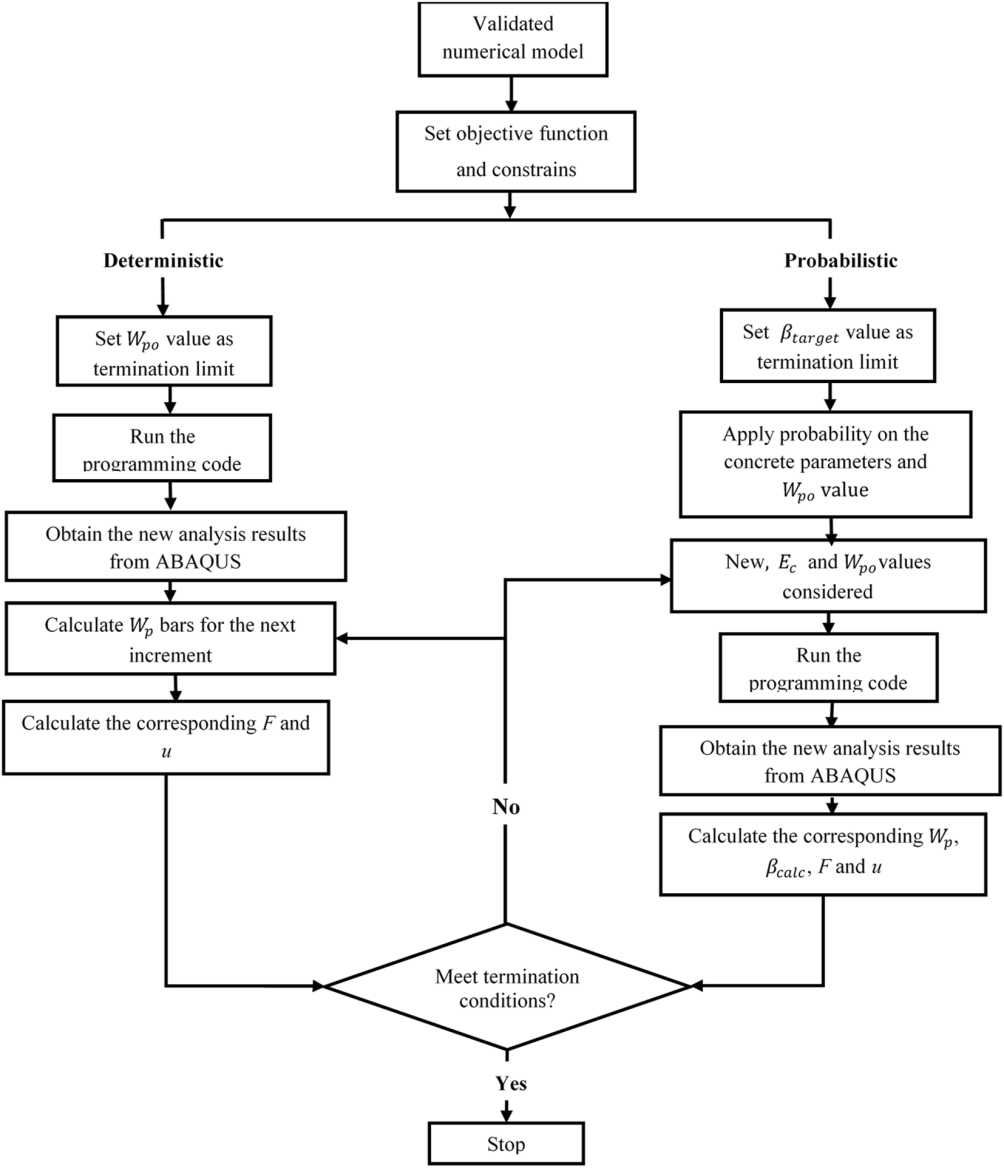
The method to resolving the optimization problem.

Where the probabilistic solution would be terminated when the calculated reliability index (*β*_*calc*_) exceeds the allowed target value (*β*_*target*_). For more illustration, this study considers the optimization steps depicted in Fig. 1, where it is important to note that the CDP parameters contained within the optimization problem are held constant.

## Model validation details

The haunched beam model in the assessment was also considered in a recent study^22^, where the same properties were taken into account in the analysis approach. This study's analysis approach started with model validation as 3D finite element models were built based on actual testing in Széchenyi István University's lab. Concrete was simulated using the Abaqus^32^ and the concrete damage plasticity (CDP) model. The numerical model was validated using the experimental results of three experimental simply supported haunched beams. Regarding the adoption testing of concrete material properties, four samples were produced for both concrete compressive behaviour using the standard cube test and tensile behaviour using the split-cylinder test. The average concrete compressive strength was found to be ${\mathop{f}\limits^{\prime}}_{c } = {27}$ MPa.

**Figure 2:**
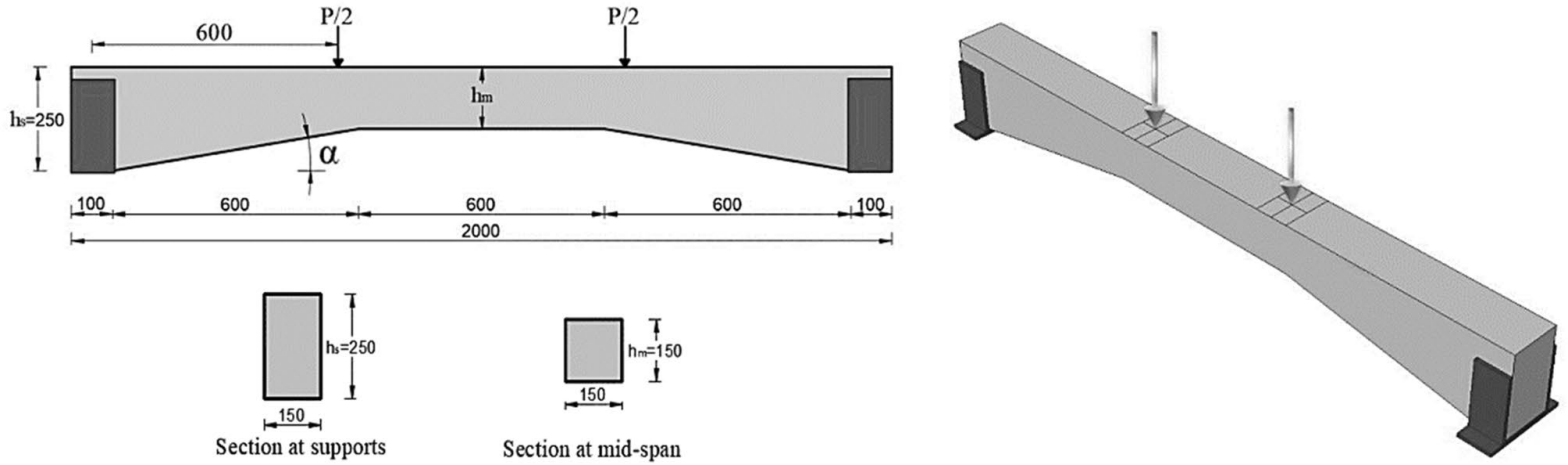
Geometry of the beams and the loading condition.

**Figure 3:**
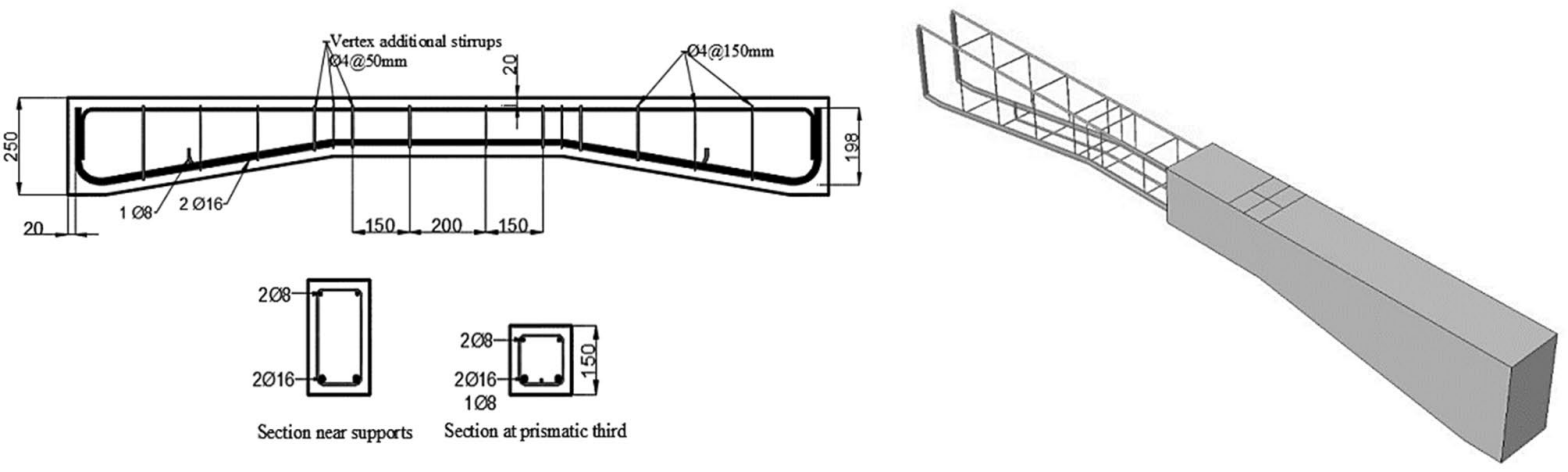
Details of reinforcement.

The haunched beams had an overall length of 2000 mm, with the cross-sectional area remaining constant at the supports but decreasing as the beam approached the mid-span, resulting in a haunch angle (α) of 9°, which was later used for reliability-based design optimization in this study. The geometry of the beams (for α of 9°), supporting conditions, and loading conditions are shown in Fig. 2. The beams were tested overall by subjecting them to two concentrated monotonic loadings till failure. Furthermore, beams have been reinforced using steel reinforcement bars of varying diameters, as shown in Fig. 3.

**Figure 4:**
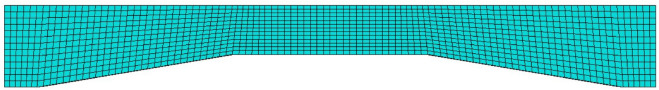
Details of the meshed model.

The finite element model provided steel reinforcement as beam elements with a 2-node linear beam in space (B31: Timoshenko beam) and concrete as an eight-node solid element (C3D8: eight-node first-order hexahedral element with accurate numerical integration). An embedded section modelled the longitudinal-transverse concrete reinforcements. Damage plasticity modelled the concrete in the haunched beams' nonlinear behaviour in finite element failure analysis, see Fig. 4.

**Figure 5:**
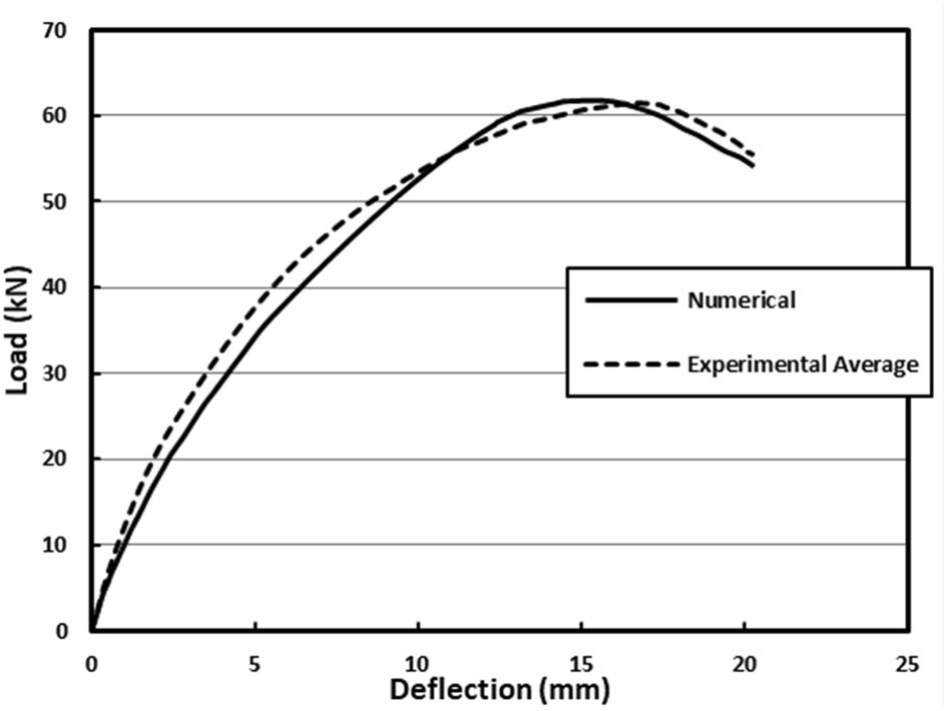
Numerical vs experimental behaviour.

Concrete damage plasticity data from specimen mechanical properties testing is imported into Abaqus to generate CDP parameters that reflect concrete damage behaviour. Compressive crushing and tensile cracking cause substantial damage to concrete. The following steps treat CDP parameters as constants. Finally, a comparison of the experimental—as an average of the three tested models- and numerical load–deflection relationship (*F*–*u)* connection is presented, as shown in Fig. 5, where the curves show that the results are compatible with each other.

To use the Reliability-based design, a standard deviation of 5% was applied to the mean values of the concrete parameters, which included a compressive strength ($\mathop f\limits^{\prime }_{{c{ }}}$) of 27 MPa and a modulus of elasticity (*E*_*c*_) of 23,500 MPa. The complementary strain energy is also considered a probabilistic value, and its mean value was extracted from each deterministic case while considering a 10% standard deviation.

## Results and discussions

This section discusses the results that were achieved by applying the proposed method to the reinforced concrete haunched beams while taking both deterministic and probabilistic solutions into consideration. In this situation, the deterministic solution was taken into consideration while *W*_*p*0_ was used as a bound (Eq. (20d)). Once the concrete properties and *W*_*p*0_ are taken into account as random variables with mean and standard deviation, the Monte Carlo method is applied to compute the reliability indices, with the total sample point number (Z = 3 × 10^8^) being taken as an assumption. Probabilistic analysis was applied while assuming that the provided reliability index functions as a bound *β*_*target*_ (Eq. (17)) Once the probabilistic analysis is applied, the reliability indices are computed.

It is important to note that the results were obtained after it was determined that the calculated deflection value (u) was less than the maximum permissible deflection value (*u*_0_ = 14.33 mm), as stated in Eq. (21a). It is also important to note that it was only after this determination that any of the results were obtained.

The complementary strain energy (*W*_*p*0_) and reliability index (*β*_*target*_) are presented in Table 1. These two variables were selected to govern the deterministic and probabilistic analyses, respectively. In addition to this, the load (*F*) that corresponds to the random concrete values (*E*_*c*_ and $\mathop f\limits^{\prime }_{{c{ }}}$) is also provided. Clearly, the concrete characteristics are randomly shifting within 5% of the mean values, and this change would unquestionably affect the related load values, which represent the uncertainty function. After receiving the data and comparing them to various scenarios, the following table presents the four possible outcomes. In any scenario, there are deterministic and probabilistic ways to approach the problem. For different median values of the complementary strain energy of the residual forces, where the *W*_*p*0_ values were taken from the four deterministic cases to produce four means ($\overline{W}_{po }$ = 0.1, 7.5, 20, 40 N.mm) with a standard deviation (*σ*_*w*_ = 10%) and a target reliability index (*β*_*target*_ = 4.9, 4.0, 3.5, 3) for every probabilistic case, where the reliability index values were selected to reflect the values presented in the Eurocode^33^.

**Table 1:** Deterministic and probabilistic results.

Case no	Case description	*W*_*p*0_ (N.mm)	*β*_*target*_	*E*_*c*_ (MPa)	${\mathop{f}\limits^{\prime}}_{c }$(MPa)	*F* (kN)
1	Deterministic	0.1	–	23,500	27	46.22
	Probabilistic		4.9	23,903	25.93	46.84
2	Deterministic	7.5	–	23,500	27	50.85
	Probabilistic		4.0	24,280	27.622	42.40
3	Deterministic	20	–	23,500	27	52.02
	Probabilistic		3.5	23,760	26.1	53.16
4	Deterministic	40	–	23,500	27	54.32
	Probabilistic		3	22,758	26.52	53.97

In general, it is possible to observe that greater loading values correlate to higher *W*_*po*_ values (in the deterministic situation) and smaller *β*_*target*_ values. This is the case because higher loading values increase the probability of failure (Probabilistic case). Because of this, the reliability of the solutions drops as the load values are higher, which will explain why the reliability index is important as a limiting value representing the probability of failure.

In addition, the purpose of complementary strain energy is made clear when damage is brought into consideration. It was mentioned that as the load increased, the values of complementary strain energy increased as well; this could be explained by the fact that as the load keeps increasing step by step, the stresses inside the steel bars increase, leading to more plastic damage in the model, as shown by the values of complementary strain energy in Table 1.

**Figure 6:**
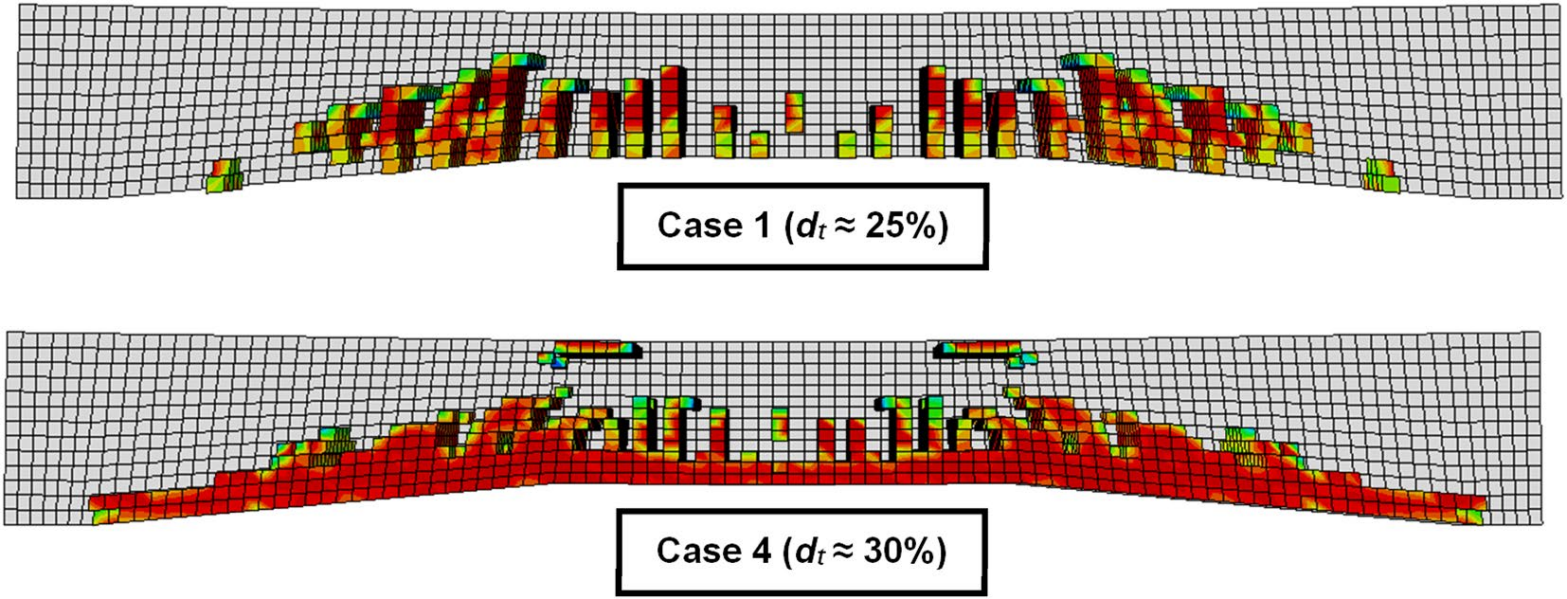
Samples of damage calculation models in two deterministic cases.

In summary, the presented results provided a detailed description of how *W*_*po*_ was applied as a bound to regulate the amount of plastic damage that occurred in the deterministic situation. At the same time, the reliability index (*β*) served as a bound in the probabilistic case of *W*_*po*_ and concrete properties being randomly changing. Table 2 provides more clarification by displaying, for each of the four scenarios, the percentage of tension damage (*d*_*t*_%) and the percentage of yielding elements (*f*_*y*_ %) in the reinforcement steel bars. On the other hand, Fig. 6 presents two deterministic examples, one with the least and one with the maximum concrete damage percentages, to illustrate and explain how the sample of the damage calculation model is taken into consideration.

**Table 2:** Damage percentages inside models.

No	Case description	*d*_*t*_ %	*f*_*y*_ %	Concrete damage intensity pattern

1	Deterministic	25.55	26.1	
	Probabilistic	25.73	25.13	
2	Deterministic	25.71	27. 92	
	Probabilistic	26.66	25.48	
3	Deterministic	27.07	29.97	
	Probabilistic	29.09	27.74	
4	Deterministic	29.26	29.42	
	Probabilistic	29.24	30.71

Table 2 demonstrates very clearly how the severity of the damage varies when comparing each case (1 to 4) or when comparing deterministic solutions to probabilistic ones.

According to the data presented in the table that is located above, higher load values are directly correlated with larger complementary strain values and more severe tension damage. The concrete damage patterns are displayed in the final column of the table based on the corresponding *d*_*t*_ % variation. This indicates that similar damage shapes were displayed when *d*_*t*_ was of a comparable in percentage. However, the severity of this pattern shifts as the percentage of damage maintained changes, with the intensity of the damage being graded from red (wholly destroyed) to blue (undamaged).

## Conclusions

Within the scope of this investigation, a probabilistic reliability-based study was carried out concerning reinforced haunched beams. This analysis took into account the fact that complementary strain energy governs the deterministic solution. In contrast, the reliability index is responsible for controlling the probabilistic solution even though the concrete parameters and complementary strain energy value are subject to random variation. This was made possible by employing Abaqus for numerical validation of the haunched beams and then linking the validated models to the author-created programming code. Accordingly, taking into consideration what has been mentioned above, the following conclusions can be drawn from the study that has been presented:

- In the deterministic solution, all the properties are considered constant; however, different values were selected, showing that higher loading values correspond to higher values; thus, more intensive damage would be observed in both concrete and steel.
- On the other hand, higher loading values correspond to smaller *β*_*target*_ values in the probabilistic case. As a result, the reliability of the solutions decreases as the load values increase, which will explain why the reliability index is useful as a limitation value reflecting the probability of failure.
- Considering values in the probabilistic case, the randomness of the probabilistic values reflects the deviation appearance within the material of the constructed structures and its impact on their behaviour. As actual structures are subjected to uncertainties, it is essential to understand the effects of such deviations on the structures' strength and damage.
- When dealing with deterministic cases, the allowable complementary strain energy *W*_*po*_ is used as a bound to regulate the amount of plastic damage that occurs. At the same time, the reliability index (*β*) served as a bound in the probabilistic scenario of *W*_*po*_ and concrete qualities being randomly changed. Both showed effectiveness in handling and limiting the plastic behaviour of the beams.
- Cases corresponding to higher load values reflect the higher complementary strain cases and own greater tension damage intensity. The concrete damage patterns depend on the corresponding percentage of tension damage *d*_*t*_% variation, indicating that similar damage shapes are shown when *d*_*t*_ is similar in percentage; however, this pattern changes in intensity.

As a result, in order to conclude, the approach that was described and the findings that were achieved provided a clear description of how the reliability index (*β*) is considered to be constrained in the situation of probabilistic randomly changed characteristics. In addition, the *W*_*po*_ value is taken to be a measurement of the plastic damage that has occurred inside the steel bars; as a result, it is taken to be bound in the deterministic solution. Thus, the limited optimal solution is provided and studied. It is also worth mentioning that the presented method is used for plastic analysis and design where the residual stresses exist, as complementary strain energy is successfully used in different types of structures and in this research, it is used as a limitation of the failure of the structure. However, out of these limitations, different mechanical experimental tests and reinforced concrete structures can be considered for any future work.

## Data Availability

The datasets generated and analyzed during the current study are available in the main manuscript; any additional details can be obtained from the Authors.
